# General developmental health in the VPA-rat model of autism

**DOI:** 10.3389/fnbeh.2013.00088

**Published:** 2013-07-24

**Authors:** Mônica R. Favre, Tania R. Barkat, Deborah LaMendola, Georges Khazen, Henry Markram, Kamila Markram

**Affiliations:** ^1^Laboratory of Neural Microcircuits, Brain Mind Institute, École Polytechnique Fédérale de LausanneLausanne, Switzerland; ^2^Department of Neuroscience and Pharmacology, Copenhagen UniversityCopenhagen, Denmark; ^3^Computer Science and Mathematics Department, School of Arts and Sciences, Lebanese American UniversityByblos, Lebanon

**Keywords:** autism, valproic acid, VPA, animal model, rat, teratogen, development

## Abstract

Autism is a neurodevelopmental condition diagnosed by impaired social interaction, abnormal communication and, stereotyped behaviors. While post-mortem and imaging studies have provided good insights into the neurobiological symptomology of autism, animal models can be used to study the neuroanatomical, neurophysiological and molecular mediators in more detail and in a more controlled environment. The valproic acid (VPA) rat model is an environmentally triggered model with strong construct and clinical validity. It is based on VPA teratogenicity in humans, where mothers who are medicated with VPA during early pregnancy show an increased risk for giving birth to an autistic child. In rats, early embryonic exposure, around the time of neural tube closure, leads to autism-like anatomical and behavioral abnormalities in the offspring. Considering the increasing use of the VPA rat model, we present our observations of the general health of Wistar dams treated with a single intraperitoneal injection of 500 or, 600 mg/kg VPA on embryonic day E12.5, as well as their male and female offspring, in comparison to saline-exposed controls. We report increased rates of complete fetal reabsorption after both VPA doses. VPA 500 mg/kg showed no effect on dam body weight during pregnancy or, on litter size. Offspring exposed to VPA 500 mg/kg showed smaller brain mass on postnatal days 1 (P1) and 14 (P14), in addition to abnormal nest seeking behavior at P10 in the olfactory discrimination test, relative to controls. We also report increased rates of physical malformations in the offspring, rare occurrences of chromodacryorrhea and, developmentally similar body mass gain. Further documentation of developmental health may guide sub-grouping of individuals in a way to better predict core symptom severity.

## Introduction

Autism is a severe and pervasive neurodevelopmental disorder, diagnosed by the age of 3 upon clinical presentation of impaired social interaction, abnormal communication, and repetitive behaviors. Autism is highly heritable (Sullivan et al., 2012), and many gene loci serve as potent risk factors (Bonora et al. in Moldin and Rubenstein, 2006; Betancur, 2011; Matsunami et al., 2013). However, the identified susceptibility genes are rare, incompletely penetrant and, often not specific to autism, and thus have not served as a direct cause of autism. In addition, environmental factors seem to play an increasingly important role, as indicated by prevalence estimates over the last 2 decades that rose from as low as 7 to as high as 72.6 cases per 10,000 (Fombonne, 2009). Importantly, broadening and improvement of the diagnostic criteria alone do not explain such an increase (Weintraub, 2011). In support of the role of the environment in autism is the accumulated evidence that biochemical insults during early embryogenesis increase the risk for autistic symptoms in the child. Examples include maternal rubella infection (Chess, 1971), ethanol (Nanson, 1992), misoprostol (Bandim et al., 2003), thalidomide (Stromland et al., 1994), and valproic acid (VPA; Clayton-Smith and Donnai, 1995; Moore et al., 2000; Ornoy, 2009; Christensen et al., 2013; Roullet et al., 2013). Thus, genetic and environmental factors seem to interact in biologically complex mechanisms to yield the broad heterogeneity in symptom severities (Zahir and Brown, 2011).

In order to elucidate the neurobiology underling autism, animal models have been developed. While an animal model may not entirely simulate a human disorder, major symptoms may be captured sufficiently to allow a rigorous study of molecular, proteomic, cellular, circuit, and behavioral alterations in order to better understand the underlying pathology. Several animal models of autism have been presented, including genotype based models affecting the oxytocin system (Modi and Young, 2012), the Reelin signaling pathway (Lakatosova and Ostatnikova, 2012; Folsom and Fatemi, 2013), the Wnt canonical pathway (Kalkman, 2012), the engrailed genes (Kuemerle et al., 2007), MeCP2 (Gonzales and LaSalle, 2010) and, neuroligins (Xu et al., 2012). Alternatively, the most used environmentally triggered model of autism results from embryonic exposure to VPA in the rat (e.g., VPA-rat model; Vorhees, 1987; Rodier et al., 1996; for review see Rodier et al., 1997; Markram et al., 2007), and a VPA-mouse model is also available (Chapman and Cutler, 1984; Wagner et al., 2006; Gandal et al., 2010; Roullet et al., 2010; Kataoka et al., 2013).

In humans, VPA is widely used as an anti-epileptic, and also prescribed as a mood stabilizer and against migraines, and it is currently in clinical trial as an anti-cancer agent. However, VPA is teratogenic (Nau et al., 1982; Ornoy, 2009), where exposure during gestation increases the risk for various congenital malformations in the child, grouped as Fetal Valproate Syndrome (FVS; Kozma, 2001; Kini, 2006; Ornoy, 2009). FVS features also include decreased verbal intelligence and an association between VPA exposure *in utero* and autism has been consistently reported (reviewed in Ornoy, 2009; Roullet et al., 2013). In particular, Moore and colleagues showed 60% of those diagnosed with FVS exhibit 2 or more autistic features, and 11% develop full blown autism (Moore et al., 2000). In the same line, Rasalam and colleagues indicated that 8.9% of children exposed to VPA *in utero* develop autistic features (Rasalam et al., 2005). Based on the nature of physical malformations and brainstem morphological changes in autism, it has become apparent that VPA exposure in the first trimester of gestation represents the highest risk for the child to develop autism, in particular after exposure during the time of neural tube closure and genesis of brainstem cranial nerve nuclei cells (Kemper and Bauman, 1998; Rodier, 2002, 2004; Arndt et al., 2005; Ornoy, 2009).

The idea of early embryogenesis as the critical time for autism led to development of the VPA rat model (Rodier et al., 1997; Kemper and Bauman, 1998; Arndt et al., 2005; Kim et al., 2011). Strong clinical validation of the model is given by observation that offspring of rats exposed to a single dose of VPA on embryonic day 12.5, around the time of neural tube closure and brain stem nuclei formation in rats, present neuroanatomical and behavioral characteristics similar to human autism. Specifically, VPA-exposed offspring present (1) a reduced number of motor cells in cranial nerve motor nuclei in the brain stem (Rodier et al., 1996; Rodier, 2002); (2) a reduced number of Purkinje cells in the cerebellum, observed both in VPA-treated offspring (Rodier et al., 1997; Ingram et al., 2000) and in autism (Kemper and Bauman, 1998); (3) decreased social interactions, increased repetitive behaviors, lower sensitivity to pain, impaired sensorimotor gating or eye blink conditioning, and increased anxiety described in VPA-treated offspring (Schneider et al., 2001, 2006, 2007; Schneider and Przewlocki, 2005; Stanton et al., 2007; Markram et al., 2008; Bambini-Junior et al., 2011; Kim et al., 2011; Zhang et al., 2012) and commonly found in the autistic spectrum (McAlonan et al., 2002; Gaigg and Bowler, 2007; Perry et al., 2007; Hofvander et al., 2009; MacNeil et al., 2009; van Steensel et al., 2011). Moreover, rat offspring treated at E12.5 exhibit strongly amplified conditioned fear memories, which generalize to non-conditioned stimuli and are resistant to extinction, in parallel to increased synaptic plasticity in the amygdala (Markram et al., 2008; see also Sui and Chen, 2012). Abnormal high anxiety, fears and phobias are also commonly reported in autism (Kanner, 1943; Muris et al., 1998; however, see Bernier et al., 2005; Evans et al., 2005; Hofvander et al., 2009; Settipani et al., 2012). Similar results have been obtained by slightly modified VPA treatment protocols in the rat (e.g., Rodier et al., 1996; Chomiak et al., 2010; Dufour-Rainfray et al., 2010; Narita et al., 2010; Kim et al., 2011; Tashiro et al., 2011), and in the mouse (e.g., Wagner et al., 2006; Gandal et al., 2010; Mehta et al., 2011; Roullet and Crawley, 2011; Hara et al., 2012; Kataoka et al., 2013). Taking into account these striking behavioral and anatomical parallels between the VPA model and human autism, the VPA rat model is well suited to study synaptic and circuit alterations in order to elucidate the potential neuropathology of autism.

The data on the VPA model not only further support a closer association between neural tube closure and autistic traits, but provide new insights into the neuropathology of autism. Studies in our lab demonstrated that the neocortex and amygdala of VPA-treated rat offspring is hyper-reactive and hyper-plastic due to hyper-connectivity between neurons (Rinaldi et al., 2007; Markram et al., 2008; Rinaldi et al., 2008a,b; Silva et al., 2009). These observations indicate excessive processing and storage of information in these nervous systems. They suggest that hyper-functional microcircuits could underlie autism, and further research is warranted.

In view of the increased use of the VPA model to study the neurobiology of autism, the goal of the present paper is to provide better practical reference to those who seek to reproduce the model. First, we describe a VPA dose dependence of offspring survival, because there is no consistent data on pregnancy outcome in the literature. We then present data on the global developmental health of VPA-treated dams and their offspring exposed to 500 mg/kg VPA during pregnancy. With this study we hope to motivate the documentation of such variables as potential predictors of autistic-like vulnerability.

## Materials and methods

### Animals and VPA-model

Outbred Wistar Han rats (Janvier Laboratories, France) were mated in house or ordered pregnant, and pregnancy was determined by the presence of a vaginal plug on embryonic day 1 (E1). To produce experimental rats based on the VPA rat model of autism (Rodier et al., 1997), we prepared sodium salt of valproic acid (NaVPA, Sigma-Aldrich) in 0.9% saline (100 mg/ml, pH 7.3). On E12.5, VPA-dams received a single intraperitoneal (i.p.) injection of NaVPA, either 500 mg/kg, 3.3 ml/kg, or 600 mg/kg, 3.3 ml/kg; control (CTR) dams received a single injection of saline vehicle (i.p., 0.9%, 3.3 ml/kg). Dams were housed individually and allowed to raise their own litters until weaning on postnatal day 23 (P23). Both male and female offspring were included in the study. The offspring were housed with 2–3 rats in same-sex, same-treatment cages. Standard plastic laboratory cages were used with bedding and *ad libitum* access to food and water, cleaned once a week. Animals were kept in a 12 h light-dark schedule with lights on at 6:30 am, in rooms under controlled humidity and temperature. All the procedures were in conformity with the Swiss National Institutional Guidelines on Animal Experimentation for the ethical use of animals, and approved by the Swiss Cantonal Veterinary Office Committee for Animal Experimentation.

### Dams and pregnancy

To determine the toxicity of different doses of VPA, we measured the complete fetal re-absorption rate (miscarriage), as the percent of dams from which a vaginal plug had been detected that did not give birth to a single pup, in CTR (*n* = 40), VPA 500 mg/kg (*n* = 26), and VPA 600 mg/kg (*n* = 37) dams. Statistical comparisons between the number of reabsorptions in each VPA treatment and the control group were done with *X*^2^ tests, and statistical significance is reported at the alpha level of 0.05.

To confirm the lower toxicity of VPA (500 mg/kg) on pregnancy, we measured the body mass of dams (CTR *n* = 9, VPA *n* = 15) on E12.5, E14, E21, P1, and, P6, for dams that gave birth to at least one pup. Analysis was done with a univariate two-way mixed factorial ANOVA (RM 2-ANOVA), using treatment (CTR or VPA) as the between subject factor, and time of weighing (E12.5, E14, E21, P1, P6) as the repeated measures within subjects factor. Statistical significance is reported at the alpha level of 0.05.

For further investigation of pregnancy outcome, the litter size was counted 2 days after birth in litters where at least one pup was born in CTR (*n* = 29) and VPA (500 mg/kg; *n* = 33). For analysis of the effect of VPA treatment on litter size, a two-tailed Student's *t*-test was applied, and statistical significance is reported at the alpha level of 0.05.

### Offspring body mass

Body mass gain of the offspring from dams treated with either vehicle (CTR) or VPA (500 mg/kg) was measured weekly for 33 postnatal weeks (PW 2–12, 15–25, and 27–38). A total of six unbalanced cohorts were evaluated (CTR *n* = 191, VPA *n* = 191), where measurement time points were inconsistent across cohorts (e.g., some cohorts measurements were from the first 12 weeks, others for months 1–7, others only for particular weeks). Thus, standard ANOVA tests were inappropriate, and we used a mixed effect model to analyse the effects of treatment (CTR or VPA) and sex (male or female) on offspring body mass over time (age of animal). The model was built using the *lmer()* function from the lme4 package in R (version 2.11.1) and, the interactions of age by sex, age by treatment, and sex by treatment were taken into account. We report |*t*-value| >2.5 to be statistically significant.

### Offspring brain mass

The brain mass of CTR (*n* = 55) and VPA (500 mg/kg, *n* = 50) offspring were measured on P1 and P14. Brains were extracted excluding the olfactory bulb, and excluding parts of the brainstem posterior to the cerebellum. A total of two balanced cohorts were used, one with paraformaldehyde (PFA) fixed and, the other with fresh brains. For the fixation procedure, animals were sedated with pentobarbital (i.p. 100 mg/kg, 100 mg/ml) and transcardially perfused with phosphate buffered saline (PBS), followed by 4% paraformaldehyde in PBS. The brains were extracted and placed in 4% PFA in PBS for 24 h, for post-fixation at room temperature, then transferred to a 30% sucrose solution in distilled water and stored at 4°C for 2–4 days until density equilibration. For fresh brain measurements, animals were removed from their home cage and decapitated, the brains dissected immediately, and placed on the weighing dish. Separate statistical analyses for P1 and P14 were done to address the effect of treatment and sex on absolute brain mass, with a univariate two-way ANOVA (2-ANOVA). *Post-hoc* analysis of the effect of treatment was carried out with Bonferroni correction for multiple comparisons. Complimentary investigation of body mass as a covariate in brain mass was done with a univariate analysis of the effect of treatment, sex, age on brain mass with a 3-way mixed model analysis of covariance (3-ANCOVA), using treatment (CTR or VPA), sex (male or female), and age (P1 and P14), as the between subject factors, and body mass as a covariate. Statistical significance is reported at the alpha level of 0.05.

### Offspring malformations

We documented any major physical malformation of the offspring from dams treated with either vehicle or 500 mg/kg of VPA, present at any time during the life of the animal. We compared the rates between the 2 treatment groups with 2-tailed FET, and report statistical significance at the alpha level of 0.05.

### Offspring chromodacryorrhea

Chromodacryorrhea is a condition found in rats due to porphyrin over-production by the Harderian glands in the eye. This exudate accumulation can form red (blood-looking) crusts around the eyes, nose, and the neck fur of the animals after grooming. Chromodacryorrhea can be used as an indicator of general stress. We documented the sign of any visible red crusts around the eyes or nose on rats, at any time during the lifetime of the animal, and compared these rates between the 2 treatment groups with 1-tailed FET. Statistical significance is reported at the alpha level of 0.05.

### Offspring olfactory discrimination behavioral test

This test measures nest-seeking behavior mediated by olfactory cues present in the home cage bedding (Gregory and Pfaff, 1971). The apparatus consisted of a clean standard housing cage (transparent polycarbonate, 375 length × 215 width × 20 height cm) with a line drawn through the center, and with two olfactory stimuli placed at opposite ends of the cage: one was a plastic Petri dish (10 cm diameter) filled with home bedding (nest odor), the other was a dish filled with clean bedding (neutral odor). On P10, male and female offspring were individually placed in the center of the cage, on the middle line, and the latency to reach the first stimulus was measured, determined by when the pup stepped with both forepaws into one of the dishes. The cage was cleaned between animals with 5% Ethanol. For clarity, statistical tests and sample sizes are specified in the results section, where statistical significance is reported at the alpha level of 0.05.

### Statistical software

Levene's tests, Student's *t*-tests, Welch's correction, 2-ANOVA and Bonferroni correction were run on GraphPad Prism software (version 4.00 for Windows, GraphPad Software, San Diego California, USA). The mixed effect linear model was run on R Freeware (version 2.11.1, www.r-project.org, ISBN 3-900051-08-9). Pearson's Chi-Square tests (X^2^), FET (used when X^2^ expected counts were bellow 5), and 3-ANCOVA were ran on IBM SPSS software (version 19 for Windows or Mac, Chicago, IL, USA).

## Results

### Dams and pregnancy

**Figure 1:**
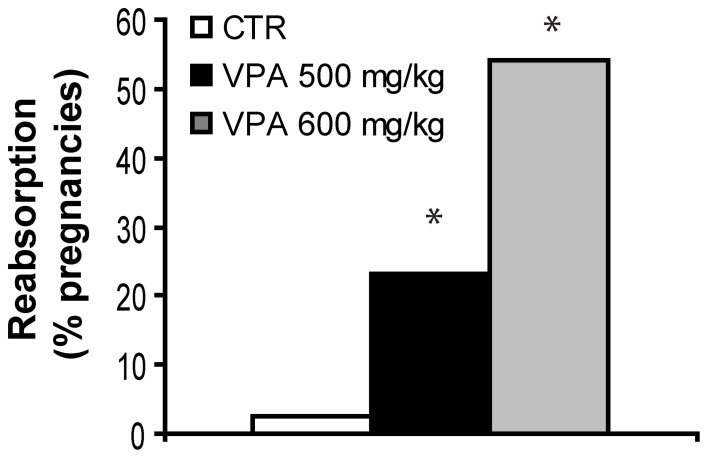
**Fetal reabsorption rates for control (CTR), VPA 500 mg/kg and, VPA 600 mg/kg**. ^*^Indicate VPA doses with higher rates than CTR. Data shown as percent of pregnancies.

The general health of both VPA-treated and control dams was normal. However, Figure 1 shows the effect of two VPA doses, 500 mg/kg and 600 mg/kg, on fetal reabsorption rates, compared with CTR. For dams with a detected vaginal plug, while 2.5% (1/40) of CTR dams showed complete fetal reabsorption, significantly higher rates were found for both VPA 500 mg/kg (23.1%, 6/26, *FET* p = 0.013 2-tailed), and VPA 600 mg/kg [54.1%, 20/37, *X*^2^_(1, 77)_ = 19.67, *p* < 0.0001] in comparison with CTR. All further studies were carried out with the VPA-treatment dose of 500 mg/kg in order to avoid a very high fetal reabsorption rate.

**Figure 2:**
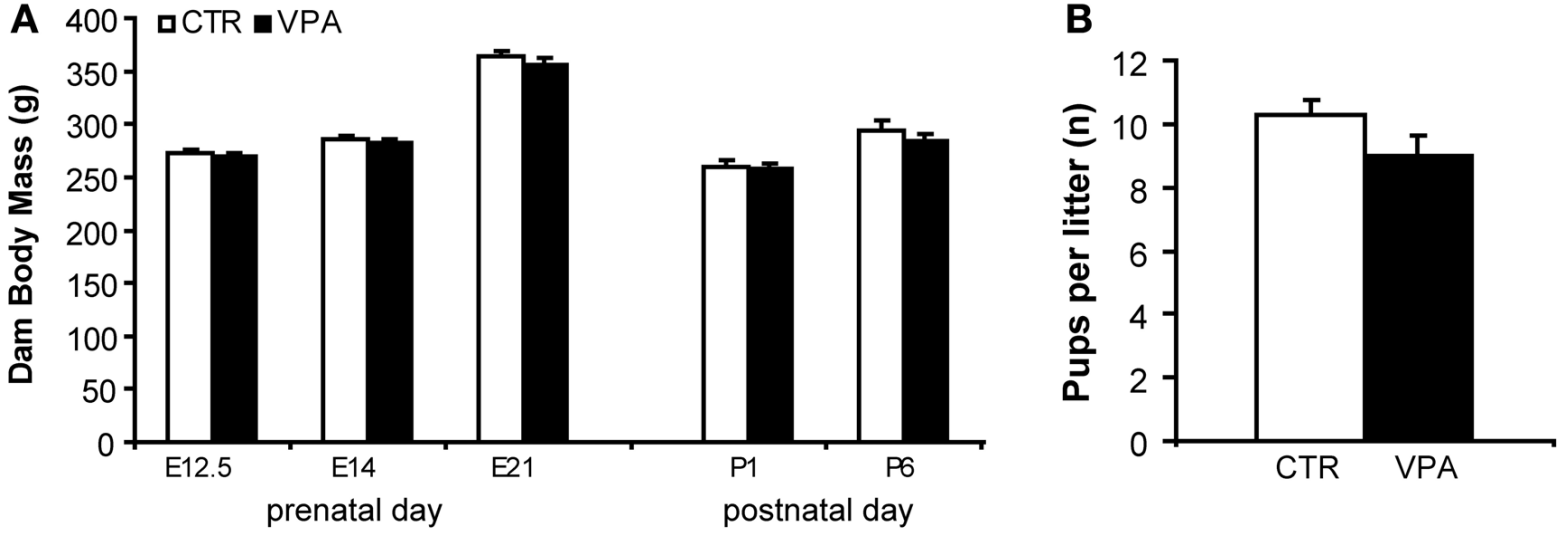
**Pregnancy outcome in control (CTR) and VPA 500 mg/kg treated dams**. **(A)** Dam body mass (grams) during embryonic days 12.5, 14, 21 (prenatal) and postnatal days 1 and 6, suffer no significant effect of treatment. **(B)** Litter size (number of pups born) is not different between treatments. Data shown as mean (*M*) and standard error of the mean (*S.E.M*.).

Figure 2A shows the effect of VPA (500 mg/kg) on pregnancy body mass compared to CTR, from measurements taken prenatally at E12.5, E14, and E21, and postpartum, on P1 and P6. We observed a significant main effect of day of weighing [*F*_(4, 97)_ = 112.3, *p* < 0.0001], but no main effect of treatment [*F*_(1, 97)_ = 2.289, *p* = 0.1335], and no interaction between treatment and day factors [*F*_(4, 97)_ = 0.2812, *p* = 0.8895]. Figure 2B shows the effect of VPA (500 mg/kg) on litter size (number of pups born) for pregnancies carried out to term (at least 1 live pup born) compared with CTR. We observed no statistically significant differences between the two treatment groups[*t*_(60)_ = 1.58, *p* = 0.118].

### Offspring body mass

**Figure 3:**
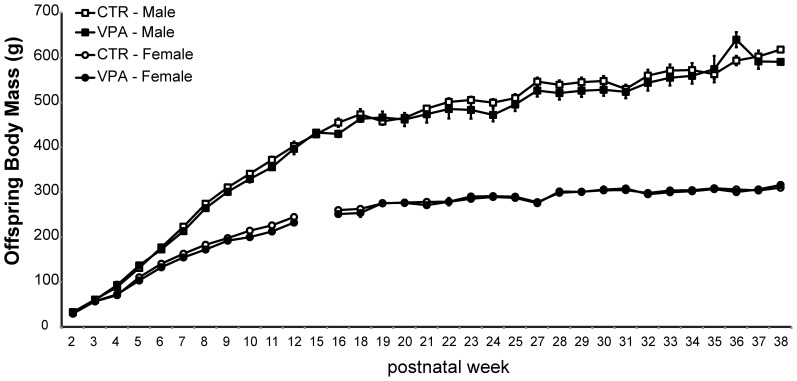
**Offspring body mass from control (CTR) and VPA 500 mg/kg groups, over 33 postnatal weeks**. Females are lighter than males but there is no effect of treatment. Data shown as mean (*M*) and standard error of the mean (*S.E.M*.).

As shown in Figure 3, prenatal treatment with VPA 500 mg/kg had no statistically significant effect on the body mass of offspring measured from postnatal weeks 2 until 38. A mixed effect model applied to assess the effect of age, sex and treatment on offspring body weight showed that the most significant contributing factor to the variability in body mass was the interaction of age by sex (age:sexMale, *t*-value = 34.47) while treatment had no effect (statusVPA, *t*-value = 0.20).

### Offspring brain mass

The brain mass of offspring treated with either CTR or VPA (500 mg/kg) is shown in Table 1, measured as absolute grams and normalized to body mass. We observed a statistically significant main effect of treatment on absolute brain mass at P1 [*F*_(1, 16)_= 16.45, *p* = 0.0009], but no main effect of sex [*F*_(1, 16)_ = 1.49, *p* = 0.241], and no interaction between sex and treatment [*F*_(1, 16)_ = 0.181, *p* = 0.676]. Bonferroni corrected *post-hoc* comparisons at P1 indicated a significantly smaller absolute brain size in VPA females (*p* < 0.05), as well as in VPA males (*p* < 0.05), in comparison to same-sex same-age CTR groups. Similar analysis repeated for P14 indicated a significant main effect of prenatal treatment [*F*_(1, 81)_ = 45.13, *p* < 0.0001], and a main effect of sex [*F*_(1, 81)_ = 4.37, *p* = 0.04], but no interaction between treatment and sex [*F*_(1, 81)_ = 0.77, *p* = 0.384]. Bonferroni corrected *post-hoc* comparisons at P14 indicated a significantly smaller absolute brain size in VPA females (*p* < 0.001), as well as in VPA males (*p* < 0.001), in comparison to same-sex same-age CTR groups.

**Table 1:** **Offspring brain mass**.

	**Sample size**		**Brain mass (g)**		**Normal. brain mass (% body g)**	
	**CTR**	**VPA**	**CTR**	**VPA**	**CTR**	**VPA**
**MALE**						
P1	5	5	0.29 ± 0.01	0.27 ± 0.01^*^	4.26 ± 0.23	3.99 ± 0.12^!^
P14	21	22	1.21 ± 0.02	1.08 ± 0.02^*^	3.93 ± 0.11	3.74 ± 0.14^!^
**FEMALE**						
P1	5	5^1^	0.27 ± 0.004	0.25 ± 0.003^*^	4.04 ± 0.26	3.98 ± 0.21^!^
P14	24	18	1.14 ± 0.41	1.01 ± 0.03^*^	4.06 ± 0.12	3.74 ± 0.16^!^

*Absolute and normalized brain mass means and S.E.M. of males or females offspring at Postnatal day 1 (P1) or 14 (P14), after embryonic exposure to saline (CTR) or valproic acid (VPA, 500 mg/kg)*.

* *, significantly different from same-sex same-age controls (CTR)*.

1 *, sample size of 4 animals for normalized brain mass*.

! *, No statistical tests were applied on normalized brain mass*.

Since the ratio of brain to body mass is not necessarily constant across age, we do not present any statistical comparisons on the normalized brain measure (a ratio), but present it for comparison to other work. We performed instead a 3-ANCOVA, where absolute brain mass means were statistically corrected for body weight (covariate). This revealed a significant main effect of prenatal treatment [*F*_(1, 95)_ = 12.86, *p* = 0.001], a significant main effect of age [*F*_(1, 95)_ = 170.14, *p* < 0.0001], but no significant main effect of sex [*F*_(1, 95)_ = 0.87, *p* = 0.35]. In addition, we found no interaction between treatment and sex [*F*_(1, 95)_ = 0.18, *p* = 0.67], but a significant interaction between treatment and age [*F*_(1, 95)_ = 6.21, *p* = 0.014] on corrected brain mass. Thus, when body mass is used as a covariate, the microencephaly effect of VPA is more prominent at P14 then at P1.

### Offspring malformations

In general, the controls as well as VPA-exposed offspring exhibited good health. However, offspring exposed to VPA 500 mg/kg occasionally exhibited some physical abnormalities including shorter snouts, multiple toes, or dwarfism (data not shown, the latter were excluded from experiments). In addition, as shown in Table 2, VPA-treated offspring presented statistically significant higher frequency of tail kinks (9% of all animals, FET *p* < 0.0001 2-tailed; 10% of males, FET *p* = 0.003 2-tailed; 9% females, FET *p* = 0.014 2-tailed), relative to CTR (0% of either sex).

**Table 2:** **Offspring tail malformations**.

	**CTR**	**VPA**
Rats (count)	162	171
Occurrences (count)	0	16
Occurrences (% of rats)	0	9^*^
Male Rats (count)	91	93
Occurrences (count)	0	9
Occurrences (% of male)	0	10^*^
Female Rats (count)	71	78
Occurrences (count)	0	7
Occurrences (% of females)	0	9^*^

*Observations after embryonic exposure to saline (CTR) or valproic acid (VPA, 500 mg/kg)*.

* *significantly different from controls (CTR)*.

### Offspring chromodacryorrhea

The frequency of occurrences of chromodacryorrhea as summarized in Table 3 represent rare events in the VPA, with a non-significant trend for a difference between CTR and VPA-exposed offspring (all animals, FET *p* = 0.07, 1-tailed; males, FET *p* = 0.133, 1-tailed; females, FET *p* = 0.523, 1-tailed).

**Table 3:** **Offspring chromodacryorrhea**.

	**CTR**	**VPA**
All Rats (count)	122	131
Occurrences (count)	0	4
Occurrences (% of rats)	0	3
Male Rats (count)	71	75
Occurrences (count)	0	3
Occurrences (% of males)	0	4
Female Rats (count)	51	56
Occurrences (count)	0	1
Occurrences (% of females)	0	2

*Observations after embryonic exposure to saline (CTR) or valproic acid (VPA, 500 mg/kg)*.

### Offspring olfactory discrimination behavioral test

**Figure 4:**
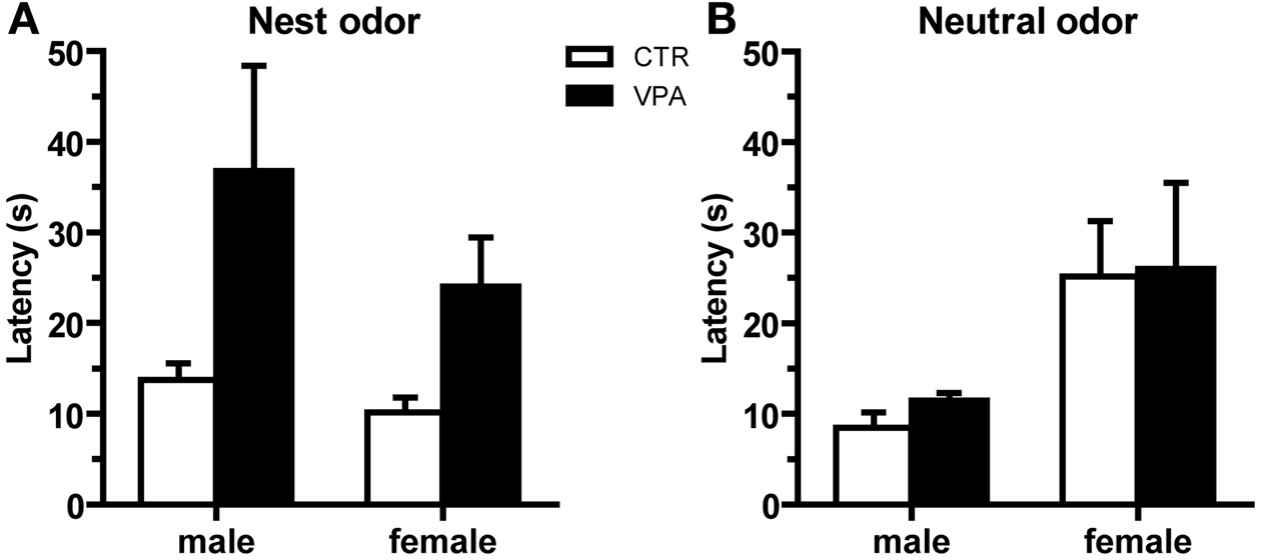
**Olfactory discrimination behavior at P10 from control (CTR) and VPA 500 mg/kg offspring**. Latencies to reach the odor of 1st choice, being either from **(A)** nest bedding, where females show an effect of treatment, and males show a trend, or from **(B)** clean bedding, where no effects were detected. Data shown as mean (*M*) and standard error of the mean (*S.E.M*.).

We assessed behavioral olfactory discrimination, where CTR and VPA 500 mg/kg males and females pups aged P10 were given a choice between nest and clean bedding. We observed no effect of treatment on the number of pups that reached the nest versus neutral odors, neither in males [CTR nest *n* = 18/22, VPA nest *n* = 18/25; *X*^2^_(1, 47)_ = 0.63, *p* = 0.43] nor in females [CTR nest *n* = 17/23, VPA nest *n* = 23/28; *X*^2^_(1, 51)_ = 0.51, *p* = 0.48]. Subsequent analysis of the latency (in seconds) to reach the nest odor when nest was as the first choice (Figure 4A), indicated that the VPA group had a significantly different variance from the CTR in males [Levene's Test *F*_(17, 17)_ = 9.841, *p* = 0.004], and in females [Levene's Test *F*_(22, 16)_ = 15.45, *p* < 0.0001]. In addition, we observed a non-significant trend for a difference between CTR and VPA in the latency to reach the nest odor in males [Welch's corrected Student *t-tests* *t*_(17)_ = 1.96, *p* = 0.067], and a significant difference in females [Welch's corrected Student *t-tests* *t*_(25)_ = 2.46, *p* = 0.021]. Lastly, Figure 4B shows the latency to reach the neutral odor when it was the first choice; we observed no differences between CTR and VPA in males [*t*_(9)_ = 1.76, *p* = 0.11], and no difference in females [*t*_(9)_ = 0.08, *p* = 0.94].

## Discussion

### Dose-dependent effects of VPA administration on pregnancy outcome

As recently reviewed by Roullet et al. (2013), clinical validity of the VPA model in rats and mice has been observed even with slight variations in the protocol for VPA administration. *In utero* exposure to VPA between 350–800 mg/kg around E11–12.5 seems to be the critical time window for most autistic-like behavioral and anatomical abnormalities in rats and mice (Ingram et al., 2000; Schneider et al., 2001; Schneider and Przewlocki, 2005; Markram et al., 2008; Kim et al., 2011). However, a precise dose threshold for specific autistic-like and non-autistic features has not been well established in the model. Interestingly, lower doses were first used to introduce abnormalities associated with *in utero* VPA exposure in the mouse (v.o. 160–100 mg/kg, multidose; Chapman and Cutler, 1984), and later, to associate *in utero* VPA exposure in the rat with autism (i.p. 300 mg/kg, single dose at E11.5, E12, E12.5; Rodier et al., 1996). For non-explicit translational reasons, subsequent studies used higher VPA doses, and demonstrated a significant increase in autistic-like behaviors in a battery of tests. For comparison, the therapeutic doses in humans currently in use range from 200 to 3600 mg/day, while the threshold for increased risks varies from 800 mg to 1400 mg/day, depending on the morphological or neurodevelopmental measure (Roullet et al., 2013). For an average female of 65 kg, these doses are equivalent to 3–55 mg/kg, a range substantially lower than that used in the current animal models (350–800 mg/kg). Importantly, species differences in the pharmacokinetics of VPA must be taken into account when interpreting the relevance of the model. For instance, VPA bioavailability in humans (70–100%) is about twice of that in rodents (34–47%;Loscher, 1999, 2002; Haddad et al., 2009; Roullet et al., 2013). Furthermore, *in utero* toxicant exposure is also likely to vary across species, but to date, few studies have focused on the issue (Binkerd et al., 1988; Hendrickx et al., 1988). As a consequence, it is difficult today to estimate how the model doses compare to humans. Nonetheless, the literature today demonstrates that VPA exposure (at presumably very high doses) in rodents at a specific time in embryonic development (neural tube closure), is sufficient to induce neurodevelopmental and morphological features that resemble autism in humans. This is an important feature of the model, because it narrows down the neural systems that are primary targets for autism vulnerability, and provides experimentally controlled conditions for the study of the genetic interactions with environmental factors that can lead to specific features. A clearer translational value of mechanisms identified in the model can emerge from future studies comparing VPA pharmacokinetics across species, and the levels of VPA required for specific deficits.

Few attempts have been made to directly compare dose effects in rodents (Wagner et al., 2006; Frisch et al., 2009). We report here that VPA administered i.p. at 500 mg/kg on E12.5 is less toxic than at 600 mg/kg in terms of fetal survival. Then, we observed no further negative pregnancy outcomes of i.p. 500 mg/kg compared to controls in terms of dam body mass, nor on the number of pups born, for pregnancies that reached term with live pups. These results complement previously published data on different strains with different doses (Binkerd et al., 1988; Stanton et al., 2007; Kim et al., 2011, 2013), and support VPA dose-dependent toxicity for fetal survival. These results in the rat-model also compliment observations in humans on dose-dependent effects of VPA on negative pregnancy outcomes, where epileptic or bipolar patients in childbearing age are recommended to use the lowest possible dose of anti-epileptics, particularly valproate (Roullet et al., 2013). In parallel, the dose of 600 mg/kg is not a practical choice for studies of autism in the offspring, due to high rates of fetal mortality. With this in mind, the scope of the current study focused on the lower dose for subsequent measures.

### Offspring body mass

There was no effect of VPA treatment on offspring body mass gain from postnatal week 2 well into adulthood (week 38). These results differ from the reduced body mass gain in VPA previously reported (Schneider and Przewlocki, 2005). This difference between the two studies may be explained by the use of 600 mg/kg males, housed five same sex animals per cage used by Schneider and Przewlocki, and difference in strain. For instance, the larger group housing may change social hierarchy and social anxiety or exercise levels, all of which could affect body weight in the Schneider and Przewlowski study but not in our case. It remains unclear if body weight observations in different versions of the VPA model are directly linked to autism-like symptoms or, if those effects are independent from autism and specific to a strain or dosage. Importantly, few studies have examined the body mass status of autistic patients (Emond et al., 2010), with inconsistent reports on the relationship with growth or obesity. While feeding symptoms and restricted diet are consistently reported to differ from controls, that is not case for energy intake which may (Evans et al., 2012) or may not (Emond et al., 2010; Kataoka et al., 2013) differ in ASD (Evans et al., 2012). Therefore, species-specific metabolic mechanisms and behavioral dietary choice or activity patters, (not modulated in standard laboratory environments for rodents, but variable for humans), all make the direct translation between the rodent model and the human body mass data currently difficult. Further research is needed on the VPA model on dietary choice and activity patterns, and their effect on growth and body mass, which may then help clarify inconsistencies in the literature.

### Offspring brain mass

We report here a reduced absolute brain mass of VPA exposed animals in early postnatal life (P1, and P14); when body mass is controlled for, the effect of VPA treatment is most apparent at P14. In contrast, absolute microencephaly (on E14 and E18) followed by macroencephaly (on P2 and P7) has been recently described in offspring of Sprague Dawley dams injected s.c. with 400 mg/kg VPA on E13 (vaginal plug counted as E1), without control for body mass (Go et al., 2012). In another variant of the VPA-model (v.o. 800 mg/kg, on E13 counted from E1 vaginal plug; Mychasiuk et al., 2012) absolute brain mass at around P100 was shown to be decreased, and there is no reference to body size. Even though body mass is expected to become less associated with brain size in adults (no longer growing), the lack of systematic presentation of absolute brain mass and, its relationship with body mass at younger ages make the effects of VPA on brain mass debatable; additional difficulty for interpretation is due to the difference in VPA administration protocols, and the lack of studies on dose effects. Thorough presentation of brain and body mass could become an important issue, as the literature in humans and other animal models suggest that autism is accompanied by premature or accelerated neuronal growth early in cortical development, which is followed by normal or reduced sizes in adolescence and adulthood (Casanova, 2007; Chomiak et al., 2010; Courchesne et al., 2011; Chomiak and Hu, 2013). Thus, additional studies are needed to determine if altered brain size and underlying causes, are predictive of more severe autistic-like abnormalities in the model. This information will also be useful to further confirm the robustness of the model in view of different protocols for model generation currently in the literature.

### Offspring physical malformations

We observed an increased occurrence of physical malformations in the tails of VPA-exposed offspring, compared to controls. These malformations indicate VPA toxicity for neural tube development. Similar results have been demonstrated in Wistar male rats exposed to 600 mg/kg VPA on E12 (Foley et al., 2012) and Sprague-Dawley male rats exposed to 600mg/kg VPA on E12 (Binkerd et al., 1988; Kim et al., 2013), further supporting at least some effects of VPA are independent of strain. These observations are also congruent with congenital malformations observed in humans exposed to VPA *in utero*, where the pattern of dysmorphic features are indicative of toxicity early in embryogenesis, around the time of neural tube closure (Rodier, 2002; Arndt et al., 2005; Tashiro et al., 2011). Birth defects in children with FVS include defects in facial features, neural tube associated, cleft pallet, cardiovascular, limb, and digits malformations, amongst others (Clayton-Smith and Donnai, 1995). In parallel, researchers have reported co-occurrence of autism and birth defects. Schendel and colleagues report 6.4% of autistic children present some form of birth defect, compared to 3.2% of children without autism (Schendel et al., 2009). Conversely, 0.43% of children presenting a birth defect presented autism, as compared to 0.22% among children without birth defects, which translates into 1.7 relative risk of presenting autism compared to children without birth defects. These were mainly isolated, and of central nervous system/eye, genitourinary, muscoskeletal, or cardiovascular nature; other defects included head/neck, respiratory, oral cleft, and gastrointestinal. Among the children with autism, male to female ratio of birth defects was approximately 7:1. Miles and colleagues report 20% of autistic patients with abnormal morphology, and 29% of these with abnormal brain morphology; they argue that full clinical morphological examination is highly sensitive to embryonic developmental insults, and thus has the power to better delineate patient subgroups (Miles and Hillman, 2000). Based on the congruence between the animal model and the human, it may be of interest to correlate birth defects with autism-like symptom severity in the models, as well as to investigate the mechanisms of VPA teratology. This knowledge may help delineate autism subgroups for clinical and genetic studies, and thus lead to better understanding of the endophenotypes more vulnerable to autistic features.

### Offspring chromodacryorrhea

We also report here noticeable, but statistically non-significant, chromodacryorrhea (“red tears”) in the VPA exposed rats, compared to none observed in controls. It remains possible however, that our sample size and analysis do not capture a true effect of VPA on rare biological contexts, and thus, that measuring chromodacryorrhea would remain indicative of vulnerable endophenotype. Chromodacryorrhea is observed due to accumulation of porphyrin after overproduction by the Harderian glands (HD) in the eye. It has been associated with physical stress (Harkness and Ridgway, 1980), joint pain (Kerins et al., 2003), bright light (Hugo et al., 1987), but not to bi-weekly or weekly home cage cleaning and human handling (Burn et al., 2006). Thus, chromodacryorrhea is used by animal caretakers as a non-invasive measure of general stress response (Burn et al., 2006; Castelhano-Carlos and Baumans, 2009). To our knowledge, no previous reports on the VPA rat demonstrated rates of chromodacryorrhea. HG secretion is related to several factors including hormonal functions, sexual differentiation, circadian rhythm, season, and age (dos Reis et al., 2005; Monteforte et al., 2009). Importantly, general stress is increased in human autism (Muris et al., 1998; Evans et al., 2005; MacNeil et al., 2009). Thus, as for physical malformations, it may be of interest to correlate the occurrence of chromodacryorrhea with autistic-like symptom severity in the model. Since stress and anxiety are frequently reported in humans (Evans et al., 2005), such studies will aid in the translational value of the VPA model.

### Offspring olfactory discrimination behavioral test

We present the VPA (500 mg/kg) olfactory deficit at P10, where females take longer to reach the nest bedding as compared to controls, and both males and females show increased variability in this latency relative to controls. The observation at P10 may result from a deficit more pronounced at younger ages, which is then not fully recovered by P10 for some individuals. In agreement with this idea, Schneider and Przewlocki (Schneider and Przewlocki, 2005) show pup olfactory discrimination of nest bedding from clean bedding was delayed in the VPA group (600 mg/kg), which took longer time than controls to reach nest bedding at P9, but recovered at P10–11. Furthermore, delayed nest seeking behavior, recovered by P11, was also observed in the VPA mouse model (Roullet et al., 2010). These observations suggest that there is a nest-seeking deficit in the VPA-exposed animals, which may be partially overcome with age in certain individuals. These observations could at first be interpreted as recovery in the older animals from an initial social deficit; however, considering it has been previously shown that the adult animals exposed to VPA *in utero* also present social behavior deficits (Roullet et al., 2013), it seems unlikely that recovery from a primary social deficit is taking place. One alternative explanation to a primary social deficit is that VPA animals may suffer from primary olfactory deficit; in this case, the observed increased VPA latencies for nest seeking would be caused by weaker or absent stimulus identification. However, such a sensory deficit would be expected to reduce the overall number of animals that reach the nest in the VPA, because more animals would seek the neutral odor in error, while this is not observed. Alternatively, VPA could suffer from a primary deficit in motor execution of the behavior. However, a generalized motor deficit would be expected to affect the latency to reach any stimulus, and this is not the case, as we observed no effect of treatment on the latency to reach the neutral bedding. On the other hand, compensatory mechanisms, such as the preference for familiar odors, may come into play with development and then guide the older animals toward the nest. Taken together, these results support a deficit in the VPA to use social-odor information to return to their nest and mother, olfactory guided survival behaviors. In addition, they support that *in utero* exposure to VPA at 500 mg/kg at E12.5 is sufficient to induce a core autism-like symptom. Future studies in the animal models are needed to determine which compensatory mechanisms may come into play with age to mask social dysfunction in certain tasks, in certain individuals. Considering social deficit is a primary symptom for autism diagnosis, such studies will not only further validate the VPA model but cue into individual differences in the level of social dysfunction in humans.

As mentioned above, while the embryonic critical time for autism vulnerability seems to be E11–12.5, there is currently no indication in the literature on which exact VPA dose produces the most valid rodent model. Our results indicate that VPA administered to the mother with a single i.p. injection of 500 mg/kg on E12.5 (counted from E1vaginal plug detection) is sufficient to induce autistic like neurodevelopmental effects. We note that cross-laboratory interpretation must be made with care, due to inconsistency in reporting how the embryonic days were counted; it is customary to treat vaginal plug detection as E1 for mice and E0 for rats, but the latter is less consistent. For instance, work in rodents that have reported E12.5 as the critical period are not inconsistent to work reporting E11.5 because only the latter counted vaginal plug day as E0. None of the reviews attempting at translating the observation in the model to autism corrected for these inconsistencies when comparing studies (Markram et al., 2007; Markram and Markram, 2010; Roullet and Crawley, 2011; Kaffman and Krystal, 2012; Modi and Young, 2012; Roullet et al., 2013).

Taking into account the striking behavioral, and anatomical parallels to human autism, the VPA-rat model allows for systematic investigations of the biological events that lead to autism-like neuropathology, from the molecular to the behavioral level, guiding new and more specific hypothesis in humans (Markram and Markram, 2010). The first studies in this direction corroborate with the idea of autism as a minicolumn-pathy (Markram et al., 2007; Markram and Markram, 2010; Williams and Casanova, 2011). Interestingly, more recent results demonstrate the overlap between genetically based models and VPA epigenetic mechanisms, supporting a potential common pathophysiological mechanism in autism (Kolozsi et al., 2009; Go et al., 2012). We present here further support of the notion that in addition to sex, the brain mass, body mass, physical malformations, and reactivity to general stress may be important covariates in future investigations of the core autistic features, and to indicate how environmental factors interact with an individual's genome to constitute particularly vulnerable endophenotype for the development of autism.

### Conflict of interest statement

The authors declare that the research was conducted in the absence of any commercial or financial relationships that could be construed as a potential conflict of interest.
